# Role and mechanism of mesenchymal stem cells in endometrial receptivity remodeling

**DOI:** 10.3389/fcell.2026.1724597

**Published:** 2026-03-12

**Authors:** Wang Zhao-Di, Liu Xian-Bao, Lv Liang-Zhen, Li Lu-Hao, Ren Jia-Jie, Zhu Hui, Jiang Bei, Chang Zhuo

**Affiliations:** 1 Heilongjiang University of Chinese Medicine, Harbin, China; 2 Yichun Central Hospital, Yichun, China; 3 The Third School of Clinical Medicine (School of Rehabilitation Medicine) of Zhejiang Chinese Medical University, Hangzhou, China

**Keywords:** embryo implantation, endometrial receptivity, infertility, mesenchymal stem cells, regenerative medicine

## Abstract

Endometrial receptivity (ER) is a pivotal determinant of successful embryo implantation, and its dysfunction is a major cause of infertility and recurrent implantation failure. Mesenchymal stem cells (MSCs) have emerged as a promising therapeutic strategy due to their multipotency, self-renewal capacity, and potent paracrine activity. This review elucidates the multifaceted mechanisms through which MSCs enhance ER, including direct differentiation into endometrial cells, promotion of angiogenesis via secretion of factors like VEGF, immunomodulation by inducing Treg cells and M2 macrophages, and remodeling of the extracellular matrix. Crucially, we highlight emerging clinical evidence; for instance, in a recent clinical trial, intrauterine infusion of umbilical cord-derived MSCs in women with intrauterine adhesions significantly increased endometrial thickness from a mean of 4.2 ± 0.5 mm to 6.8 ± 0.7 mm and improved the clinical pregnancy rate to 38.5%. Furthermore, we discuss ongoing clinical trials and future directions, such as the development of engineered MSC-derived exosomes and biomaterial-scaffold combinations. Despite challenges in standardization and long-term safety, MSC-based therapy represents a novel and potent approach for regenerating dysfunctional endometrium, offering new hope for refractory infertility.

## 1 Introduction

Endometrial receptivity (ER) refers to a transient, precisely regulated period during the menstrual cycle when the endometrium acquires a functional phenotype capable of supporting blastocyst apposition, adhesion, and invasion (Craciunas et al., 2019). The synchronization between a viable embryo and a receptive endometrium is indispensable for successful implantation (Lessey and Young, 2019). In clinical practice, despite the transfer of high-quality embryos, a significant proportion of patients undergoing *in vitro* fertilization (IVF) experience implantation failure due to compromised ER (Somigliana et al., 2023). Current treatment options for impaired ER, such as hormonal supplementation, often yield suboptimal outcomes. Mesenchymal stem cells (MSCs), with their self-renewal, multidirectional differentiation, and potent immunomodulatory and paracrine capacities, have emerged as a promising therapeutic strategy for endometrial regeneration (Rendra et al., 2020). To compile this narrative review, we conducted a comprehensive literature search in PubMed, Web of Science, and Embase databases from inception to December 2024. The search strategy employed combinations of keywords including “mesenchymal stem cells,” “endometrial receptivity,” “endometrial regeneration,” “intrauterine adhesions,” “thin endometrium,” “recurrent implantation failure,” “angiogenesis,” and “immunomodulation.” We prioritized peer-reviewed original research articles (both preclinical and clinical), systematic reviews, and clinical trials published in English. While previous reviews have discussed MSCs in reproductive medicine, this article uniquely integrates the multifaceted mechanistic actions of MSCs—direct differentiation, paracrine-mediated angiogenesis, immunomodulation, and microenvironmental remodeling—with emerging clinical evidence and critically examines the current translational bottlenecks. This integrated perspective aims to bridge the gap between bench-side discoveries and bedside applications, providing a roadmap for future research and clinical translation. This review aims to systematically summarize the latest advances in the mechanisms of MSCs in restoring ER, critically evaluate emerging clinical evidence, and discuss current challenges and future translational perspectives.

## 2 Overview of mesenchymal stem cells

### 2.1 Sources and characteristics of mesenchymal stem cells

Mesenchymal stem cells (MSCs) can be isolated from a variety of tissues, each source conferring distinct advantages and limitations for clinical application. While they share core characteristics—such as adherence to plastic, specific surface marker expression (e.g., CD73^+^, CD90^+^, CD105+), and multilineage differentiation potential—the choice of source significantly impacts cell yield, proliferative capacity, and paracrine profile, which in turn influences their therapeutic efficacy for endometrial repair. To provide a clear comparison, the key features of MSCs from predominant sources are summarized in Table 1.

**TABLE 1:** Characteristics of MSCs from different sources and their potential in endometrial repair.

Source	Key advantages	Key limitations	Preclinical/Clinical evidence (examples)
Bone Marrow MSCs (BMSCs)	• Most extensively studied, well-defined biology • Strong proliferative and multidifferentiation capacity	• Invasive harvesting procedure, donor morbidity • Cell number and activity decline with age	• Increased endometrial thickness and gland number in rat thin endometrium models (Jing et al., 2014) • Widely used as “seed cells” in tissue engineering
Umbilical Cord MSCs (UCMSCs)	• Non-invasive source, ethically unproblematic • High proliferative potential, low immunogenicity • Suitable for “off-the-shelf” cell banks	• Variable initial cell yield upon isolation • Potential heterogeneity between donors	• A Phase I clinical trial showed safety and efficacy for severe IUA using UC-MSCs on collagen scaffolds (March, 2011) • Rich secretome (VEGF, HGF) promotes angiogenesis (Wang et al., 2013)
Placental MSCs (PMSCs)	• Abundant tissue source, high cell yield • Unique immunosuppressive properties	• Immunomodulatory potency may be lower than BMSCs (Dai et al., 2023) • Less studied, lack of standardization	• Demonstrated high efficacy in repairing endometrial damage *in vivo* and *in vitro* (Liu et al., 2024) • Polarizes macrophages toward anti-inflammatory M2 phenotype (Fazekasova et al., 2011)
Adipose-derived MSCs (ADMSCs)	• Abundant source, minimal morbidity (liposuction) • Potent paracrine function	• Lower osteogenic potential • Cell characteristics may be influenced by donor BMI	• Upregulated angiogenic factors and improved perfusion in thin endometrium animal models (Wang et al., 2021) • Secretes IGF-1, TGF-β, stimulating endometrial cell proliferation and ECM remodeling (Fiedler et al., 2018)

Following the comparison in Table 1, it is evident that the diverse properties of MSCs from different origins provide a versatile toolkit for addressing various pathological aspects of impaired endometrial receptivity. For infertility caused by a compromised endometrium, MSCs hold the potential to precisely “repair” and “remodel” the damaged tissue through their self-renewal capacity, multidirectional differentiation potential, and robust paracrine activity. By fundamentally optimizing the endometrial microenvironment—promoting angiogenesis, modulating immune status, and regulating extracellular matrix remodeling—MSCs can create ideal conditions for embryo implantation.

### 2.2 Methods of identification of MSCs

#### 2.2.1 Cell morphology observation

Under the light microscope, MSCs usually present a fibroblast-like morphology, and the cells are generally spindle or spindle-shaped with elongated cytosol and prominent cytoplasmic protrusions. These morphological features usually remain relatively stable during cell culture and provide a basic basis for their morphological characterization (Dai et al., 2023). However, as the number of cell passages increases or the culture conditions change, these morphological features may change to some extent. For example, during high-generation passages, cells may exhibit morphological changes such as flattening and enlargement. At this time, the proliferative capacity of the cells may show a decrease and even the functional characteristics may change to some extent, which means that their self-renewal capacity and differentiation potential may be affected (Hematti, 2012).

Such changes suggest that purely morphological features alone are not sufficient to fully assess the status of cells when performing morphological observations. Instead, it is particularly important to combine the cell background, passaging history, and culture conditions to make a comprehensive judgment. In summary, the morphological features of cells are only part of the assessment of their status, how to combine with other indicators, such as proliferation ability, differentiation potential and molecular markers, in order to more accurately judge the quality and function of cells.

#### 2.2.2 Surface marker detection

Currently, MSCs still do not possess uniform specific markers and lack standardized identification methods. One way to characterize it is to detect its specific surface markers. Usually, MSCs exhibit a series of characteristic markers, such as CD73, CD90, and CD105, while showing low or no expression for CD34 and CD45 (Denu et al., 2016). The detection of these surface markers can provide researchers with important biological information to confirm the identity and characterization of cells. To achieve this purpose, techniques such as flow cytometry, immunofluorescence staining and immunohistochemistry are widely used. Flow cytometry, due to its high throughput, rapidity and accuracy, has become one of the most commonly used methods to identify surface markers of MSCs. It is capable of simultaneously detecting the expression of multiple markers and quantitatively analyzing cell populations (Lin et al., 2013). For example, in the identification of bone marrow MSCs, it was found by flow cytometry that the positive expression rates of CD73, CD90, and CD105 were usually high, while the expression rates of CD34 and CD45 were extremely low, which was consistent with the characteristics of MSCs. In contrast, immunofluorescence staining can visualize the expression and localization of markers at the cellular level, thus revealing the distribution of markers on the cell surface. In particular, immunofluorescence staining provides an extremely effective tool for studying the co-expression of cellular markers and their specific distribution in different cell types. It can not only reveal the surface characteristics of cells, but also reflect the changes in the cellular state through the strength and distribution of fluorescent signals (Corselli et al., 2013).

#### 2.2.3 Identification of multidirectional differentiation potentials

MSCs have significant multidirectional differentiation potential and can differentiate into many different types of cells, including osteoblasts, adipocytes, and chondrocytes (Schieker et al., 2004). In order to identify their osteogenic differentiation ability, alizarin red staining is often used for detection. After inducing the differentiation of MSCs to osteoblasts, the cells will form mineralized nodules, and after alizarin red staining, these nodules will show red color, thus indicating that the cells have successfully differentiated into osteoblasts (Luzzani and Miriuka, 2017). For the identification of adipogenic differentiation, oil red O staining is usually used. After induced differentiation of adipocytes, a large number of lipid droplets will accumulate in the adipocytes, and these lipid droplets will appear red under oil red O staining, so that the successful differentiation into adipocytes can be confirmed by this method (Zainal Ariffin et al., 2024). The identification of chondrogenic differentiation is mostly accomplished by Alcian blue staining, where chondrocytes synthesize glycosaminoglycans, such as chondroitin sulfate, during differentiation, and these substances, when combined with the Alcian blue dye, show blue color, suggesting that the differentiation process of chondrocytes has occurred (Alonso-Perez et al., 2022). These staining techniques provide us with a visual way to observe and confirm the multidirectional differentiation potential of MSCs. In order to ensure the accuracy and reliability of the identification results, it is necessary to operate in strict accordance with the induction conditions in the experimental protocol to ensure the stability and consistency of the experiment, so as to draw scientific and reliable conclusions.

## 3 Factors affecting endometrial receptivity and methods of assessment

### 3.1 Factors affecting endometrial receptivity

Endometrial receptivity is finely regulated by multiple interrelated factors (Lu et al., 2017). Understanding these determinants is essential for elucidating how MSCs exert their therapeutic effects and for guiding targeted interventions in infertility (Sun et al., 2024) (Ochoa-Bernal and Fazleabas, 2020).

#### 3.1.1 Endometrial thickness and morphology

Endometrial thickness and morphology serve as the structural “gateway” for embryo implantation. During the window of implantation, an optimal endometrial thickness of 7–12 mm provides adequate physical support for embryo apposition and facilitates trophoblast invasion (Achache and Revel, 2006). Ultrasonographically, a receptive endometrium typically exhibits a “triple-line” pattern, reflecting orderly tissue architecture conducive to embryo localization and adhesion (Wang Y. et al., 2018). Conversely, structural abnormalities—whether insufficient thickness, disrupted layering, or focal pathology—compromise this physical scaffold, impeding embryo positioning and failing to provide stable attachment (Munro, 2019). These structural deficits represent a fundamental rationale for MSC-based therapy. Unlike hormonal treatments that merely stimulate existing tissue, MSCs can directly regenerate atrophic or damaged endometrium through two complementary mechanisms: (1) differentiation into endometrial epithelial and stromal cells to physically replace lost tissue, and (2) paracrine secretion of growth factors that promote proliferation of residual endometrial cells. This dual action addresses the “structural gateway” deficit at its source, restoring the physical architecture necessary for embryo implantation (detailed in Section 3.1).

#### 3.1.2 Hormone level

Ovarian steroid hormones orchestrate endometrial dynamics with precision. During the follicular phase, estrogen drives endometrial proliferation, progressively increasing thickness and glandular complexity (de Ziegler et al., 1998). Following ovulation, progesterone induces secretory transformation: endometrial glands secrete nutrients essential for early embryonic nutrition, while stromal cells undergo decidualization, creating a receptive endometrial bed (Young, 2013). Pathological hormonal imbalances disrupt this process. In premature ovarian insufficiency, estrogen deficiency restricts endometrial proliferation (Yu et al., 2022). Conversely, unopposed estrogen exposure leads to endometrial hyperplasia, paradoxically reducing receptivity (Jankowska, 2017). Androgens modulate endometrial energy metabolism; hyperandrogenemic states downregulate receptivity-associated genes and impair endometrial function (Lissaman et al., 2023) (Yusuf et al., 2023). Thyroid hormone and prolactin exert additional modulatory effects through systemic metabolic regulation (Binita et al., 2009) (Kakita-Kobayashi et al., 2020).

Crucially, MSCs operate synergistically with—rather than independently of—this hormonal milieu. *In vitro* studies demonstrate that MSCs express functional hormone receptors (estrogen receptor ER, progesterone receptor PR) and respond to hormonal cues in the microenvironment, guiding their differentiation toward endometrial lineages (Zheng et al., 2018). Moreover, MSC paracrine factors may help restore hormonal responsiveness in pathologically altered endometrial cells, potentially reversing conditions like progesterone resistance. This hormone-responsive property distinguishes MSCs from purely mechanical interventions and underpins their ability to integrate with, rather than override, the body’s physiological regulatory systems.

#### 3.1.3 Immune factors

The endometrial immune microenvironment functions as a critical “security checkpoint” during implantation. Under physiological conditions, uterine natural killer (uNK) cells promote vascularization through secretion of VEGF and placental growth factor (Acar et al., 2011). Concomitantly, regulatory T cells (Tregs) establish local immune tolerance, preventing rejection of the semi-allogeneic embryo.

Pathological immune dysregulation disrupts this equilibrium. In conditions associated with recurrent implantation failure, uNK cell function becomes aberrant, potentially exerting excessive cytotoxicity against trophoblast cells (Sfakianoudis et al., 2021). Cytokine profiles shift from tolerogenic (TGF-β, IL-10) toward pro-inflammatory (TNF-α, IFN-γ), creating a “hostile” endometrial microenvironment (Xie et al., 2022). This immune disequilibrium provides perhaps the most compelling rationale for MSC-based immunotherapy. As detailed in Section 4.3, MSCs possess potent immunomodulatory capabilities that precisely target these very mechanisms: they induce expansion of Tregs through TGF-β and PGE2 secretion, polarize macrophages from pro-inflammatory M1 toward anti-inflammatory M2 phenotypes, and directly inhibit aberrant uNK cell cytotoxicity. Through this coordinated immunomodulation, MSCs can potentially convert a “hostile” immune microenvironment into a tolerogenic one conducive to embryo implantation—an effect unachievable with conventional hormonal therapy alone.

#### 3.1.4 Blood perfusion

Adequate endometrial perfusion constitutes the “lifeline” for implantation and early embryonic development. The uterine artery and its branches deliver oxygen and nutrients while removing metabolic waste, maintaining endometrial cellular metabolism and functional integrity (Zhang et al., 2020). During the window of implantation, angiogenesis intensifies under the influence of VEGF and other factors, creating a dense microvascular network at the anticipated implantation site (Krussel et al., 2003). In pathological states—notably endometriosis and chronic endometritis—inflammatory vascular damage and vasospasm increase uterine artery resistance indices, severely compromising endometrial perfusion (Xavier et al., 2005). The resultant hypoxia and nutrient deprivation downregulate expression of key receptivity molecules, rendering even structurally normal endometrium functionally deficient (Ke et al., 2023). The potent pro-angiogenic properties of MSCs offer a direct therapeutic strategy to address this vascular insufficiency. MSCs secrete a rich cocktail of angiogenic factors—including VEGF, bFGF, and HGF—that act synergistically to stimulate endothelial proliferation, migration, and tube formation. Unlike pharmacological vasodilators that provide temporary flow increases, MSC-mediated angiogenesis rebuilds the endometrial microvasculature structurally, restoring durable perfusion. Preclinical studies consistently demonstrate that following MSC transplantation, VEGF expression is significantly upregulated and microvessel density markedly increases in the endometrium (Yang et al., 2011), providing the vascular “lifeline” essential for embryonic development (discussed in Section 4.3).

### 3.2 Assessment methods of endometrial receptivity

Accurate assessment of endometrial receptivity is essential for optimizing infertility treatment and critically, for evaluating therapeutic responses to MSC-based interventions. Current assessment modalities approach receptivity from complementary structural, histological, and endocrine perspectives.

Ultrasound evaluation serves as the first-line clinical tool due to its convenience and non-invasiveness. Transvaginal ultrasonography enables clear visualization of endometrial thickness, pattern, and hemodynamics (Li et al., 2023). During the window of implantation, endometrial thickness (optimally 7–12 mm) and the presence of a clear “triple-line” pattern suggest receptive morphology (Wu et al., 2022). Color Doppler enhances assessment by quantifying uterine artery and subendometrial blood flow parameters: lower resistance index (RI) and pulsatility index (PI) values correlate with better perfusion and enhanced implantation potential (Zhang et al., 2022; Cheng et al., 2022). For MSC therapy monitoring, serial ultrasound assessments provide non-invasive, real-time measures of structural improvement—increased thickness, restored layering, and enhanced vascularity—serving as practical surrogate markers of therapeutic efficacy. Endometrial biopsy represents the invasive gold standard for receptivity assessment. Histological evaluation during the window of implantation reveals secretory transformation—tortuous glands with luminal secretions and stromal decidualization—confirming appropriate developmental synchronization (McCluggage, 2006). Biopsy also enables detection of pathology (chronic endometritis, fibrosis) and molecular analyses (receptivity gene arrays, cytokine profiling). In the context of MSC therapy, post-treatment biopsies offer definitive evidence of cellular and functional restoration: immunohistochemistry can confirm donor-cell integration, while molecular analyses can document normalization of receptivity-associated gene expression—providing mechanistic confirmation beyond structural improvement.

Hormonal assays provide endocrine insight into endometrial preparation. Serum estradiol (E_2_) and progesterone (P) measurements track endocrine progression; mid-luteal P/E_2_ ratio abnormalities suggest inadequate secretory transformation (Paulson, 2019). However, hormone levels exhibit considerable variability and incompletely reflect the complex local endometrial microenvironment, necessitating integration with other assessment modalities.

Critically, these assessment modalities serve dual purposes: they establish baseline diagnosis before intervention and function as essential tools for monitoring therapeutic response afterward. The multidimensional integration of ultrasound parameters (structural improvement), histological/molecular analyses (cellular and functional restoration), and clinical outcomes (pregnancy and live birth rates) will be crucial for objectively evaluating MSC efficacy, optimizing treatment protocols, and identifying patient subgroups most likely to benefit in future clinical trials.

## 4 Mechanisms by which mesenchymal stem cells improve endometrial tolerance

**FIGURE 1:**
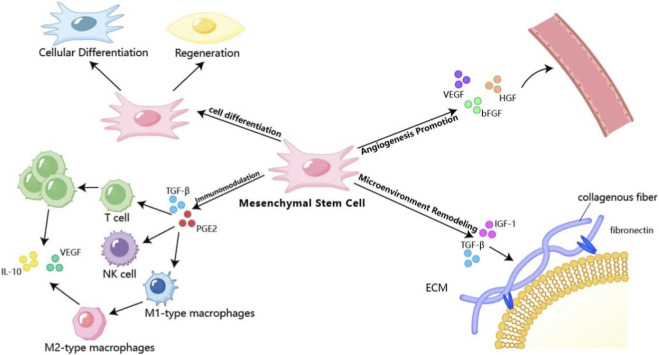
Schematic diagram of the multifaceted mechanisms by which MSCs improve endometrial receptivity.

Before detailing the specific mechanisms, a conceptual distinction warrants emphasis: endometrial repair and functional receptivity are related but not synonymous. Structural repair—manifesting as increased endometrial thickness, restored glandular epithelium, enhanced vascular density, and reduced fibrosis—represents the foundation upon which receptivity is built. However, true functional receptivity requires more than structural integrity; it demands the precise temporal and spatial orchestration of hormonal responsiveness, immune tolerance, and embryo-endometrial cross-talk that characterizes the window of implantation. This distinction carries important implications for interpreting therapeutic outcomes: improvements in ultrasound parameters or histology following MSC treatment provide evidence of successful tissue repair, but do not automatically guarantee restoration of functional receptivity. The following sections detail how MSCs contribute to both domains: they facilitate structural regeneration through differentiation and angiogenesis (Section 4.1; Section 4.2), while simultaneously orchestrating the functional maturation of the endometrial niche through immunomodulation and microenvironmental remodeling (Section 4.3; Section 4.4). Both dimensions are necessary, but only their integration achieves the ultimate goal—successful embryo implantation and live birth. Mesenchymal stem cells (MSCs) ameliorate impaired endometrial receptivity through a multifaceted and synergistic approach, which can be categorized into four core mechanistic themes: cellular differentiation, paracrine-mediated angiogenesis, immunomodulation, and microenvironmental remodeling. These interconnected pathways collectively restore the structural and functional integrity of the endometrium, creating an ideal niche for embryo implantation. The schematic illustration of these mechanisms is presented in Figure 1.

### 4.1 Differentiation into endometrial cells

MSCs possess the capacity to directly differentiate into endometrial cell lineages, thereby contributing to the structural regeneration of damaged tissue. This functional plasticity enables them to serve as a cellular reservoir for replacing lost or dysfunctional endometrial epithelial and stromal cells (Xia et al., 2019).

#### 4.1.1 *In vivo* experimental evidence

Numerous animal experiments have strongly confirmed the potential of MSCs to differentiate into endometrial cells *in vivo* and their role in repairing and improving the structure and function of the endometrium. Zhao et al. constructed a rat model of endometrial injury and transplanted labeled bone marrow MSCs into the animal model by intrauterine injection. Subsequent observations using immunohistochemical staining techniques revealed that donor-derived MSCs could differentiate into cells expressing endometrial epithelial cell markers (e.g., cytokeratins) and stromal cell markers (e.g., vimentin) within the damaged endometrial areas (Jing et al., 2014). These differentiated cells integrated into the endometrial structure, participating in tissue reconstruction, which resulted in significantly increased endometrial thickness, restored gland number, and improved blood supply, thereby enhancing receptivity (Jiang et al., 2017). Similar studies in other animal models, such as mice and rabbits, have corroborated these findings, demonstrating the universality of MSC differentiation and endometrial repair *in vivo* (Wang and Tan, 2023).

#### 4.1.2 *In vitro* experimental evidence

*In vitro* induced differentiation experiments provide a controlled platform for investigating the process and mechanism of MSC differentiation. Zheng et al. simulated the endometrial microenvironment using an induction medium containing specific factors like estradiol, epidermal growth factor (EGF), transforming growth factor-β (TGF-β), and platelet-derived growth factor BB (PDGF-BB) (Zhang et al., 2012). Under these conditions, MSCs underwent morphological changes from a spindle shape to a polygonal shape resembling endometrial epithelial cells. Molecular analyses, including immunohistochemistry, PCR, and Western blot, confirmed the high expression of endometrial epithelial cell-specific markers (e.g., keratin, estrogen receptor ER, progesterone receptor PR) and stromal markers (e.g., vimentin) (Zheng et al., 2018). Functional assays further demonstrated that the differentiated cells exhibited enhanced adhesion to embryonic trophoblast cells and secreted cytokines relevant to receptivity under hormonal stimulation, strongly supporting the feasibility of MSC differentiation into endometrial cells *in vitro* (Meng et al., 2007).

### 4.2 Promote angiogenesis

Adequate vascularization is fundamental to endometrial receptivity, ensuring the delivery of oxygen, nutrients, and growth factors essential for the developing embryo. MSCs are potent instigators of angiogenesis, primarily through their rich secretome of pro-angiogenic factors (Alfer et al., 2017).

Vascular endothelial growth factor (VEGF) is a central mediator, which binds to its receptors (VEGFR-1/2) on endothelial cells, activating key signaling pathways like PI3K/Akt and MAPK to stimulate endothelial cell proliferation, migration, and the formation of new vascular branches (Tsuzuki et al., 2013; Hall and Hubbell, 2004; Gerber et al., 1998; Lin et al., 2010; Chen et al., 2017). *In vivo* studies consistently report that following MSC transplantation, VEGF expression is significantly upregulated in the endometrium, accompanied by a marked increase in microvessel density (MVD), providing direct evidence for MSC-driven angiogenesis (Yang et al., 2011). Other factors, including basic fibroblast growth factor (bFGF) and hepatocyte growth factor (HGF), act synergistically with VEGF. bFGF enhances endothelial cell mitosis and facilitates cell migration by remodeling the extracellular matrix, while HGF promotes both endothelial proliferation and vessel maturation by regulating pericyte interactions, ensuring the formation of stable and functional vasculature (Yu et al., 2017; Wang L. S. et al., 2018). While preclinical studies consistently demonstrate MSC-induced angiogenesis through VEGF and other factors, several critical considerations warrant acknowledgment. First, the majority of evidence derives from rodent models, whose endometrial vascular biology and cyclic remodeling differ substantially from humans; extrapolation to clinical settings requires caution. Second, the paracrine secretion of pro-angiogenic factors by MSCs is inherently transient, raising unanswered questions about whether sustained vascular remodeling requires repeated administration or engraftment of cells capable of long-term persistence. Third, increased microvessel density—the most commonly reported outcome—serves as a surrogate marker rather than direct evidence of functional perfusion. Whether angiogenic improvements translate to enhanced nutrient delivery at the embryo-endometrial interface remains incompletely demonstrated. Finally, it remains unclear whether the magnitude of angiogenesis induced by MSCs in preclinical models is clinically sufficient to overcome the perfusion deficits characteristic of conditions like thin endometrium or intrauterine adhesions. These gaps underscore the need for studies employing advanced perfusion imaging and correlating vascular outcomes with pregnancy rates in appropriately powered clinical trials.

### 4.3 Regulation of immune response

MSCs critically modulate the local immune microenvironment, shifting it from a pro-inflammatory state to an immune-tolerant milieu that facilitates embryo implantation. This immunomodulation is achieved by coordinately regulating both innate and adaptive immune cells (Pusztaszeri et al., 2006). A pivotal mechanism involves the expansion of regulatory T cells (Tregs). MSC-derived soluble factors, notably transforming growth factor-β (TGF-β) and prostaglandin E2 (PGE2), induce the differentiation and proliferation of Tregs (Robertson et al., 2022; Kelly et al., 2017). These Tregs subsequently suppress effector T-cell activity and secrete anti-inflammatory cytokines like interleukin-10 (IL-10), which is essential for establishing maternal-fetal tolerance and preventing excessive immune attack on the semi-allogeneic embryo (Noh et al., 2016; Moffett and Colucci, 2014).

Concurrently, MSCs exert profound effects on innate immune cells. PGE2 secreted by MSCs can directly inhibit the cytotoxicity of uterine natural killer (uNK) cells, thereby reducing their potential to harm invading trophoblast cells (Thuere et al., 2007). Furthermore, MSCs drive the polarization of macrophages from the pro-inflammatory M1 phenotype towards the anti-inflammatory and tissue-reparative M2 phenotype (Schumacher et al., 2012). M2 macrophages contribute to resolving inflammation and promoting vascularization by secreting factors such as IL-10 and VEGF, thereby creating a favorable environment for implantation and tissue healing (Yamashita et al., 2015).

In summary, through this coordinated regulation of T cells, NK cells, and macrophages, MSCs orchestrate a comprehensive immunomodulatory network that is fundamental to restoring endometrial receptivity in cases of immune dysregulation. Despite compelling *in vitro* and animal data supporting MSC-mediated immunomodulation, the clinical evidence in the context of endometrial receptivity remains preliminary and faces substantial translational hurdles. Most studies have focused on quantifying immune cell populations (e.g., Tregs, M2 macrophages) in peripheral blood or endometrial biopsies, rather than demonstrating functional immune tolerance at the maternal-fetal interface—a conceptually distinct endpoint. The heterogeneity of immune dysfunction among patients with recurrent implantation failure or intrauterine adhesions suggests that not all individuals may benefit equally from MSC immunotherapy; Some may have predominant uNK cell abnormalities, others Treg deficiencies, and still others mixed patterns. The absence of validated immune biomarkers to prospectively identify responders versus non-responders represents a critical gap limiting clinical implementation. Furthermore, the optimal timing of MSC administration relative to embryo transfer—whether immunomodulation requires weeks to establish or can be achieved acutely—remains undefined. Future research should prioritize longitudinal immune profiling in clinical trial participants to elucidate these variables and enable personalized immunomodulatory approaches.

### 4.4 Regulation of endometrial microenvironment

Beyond direct cellular interactions, MSCs orchestrate a holistic remodeling of the endometrial microenvironment, fine-tuning the extracellular matrix (ECM) and critical signaling pathways to optimize the functional state of the endometrium.

#### 4.4.1 Extracellular matrix remodeling

The extracellular matrix (ECM) provides crucial physical and biochemical support for endometrial cells and embryo implantation. MSCs precisely regulate ECM synthesis and degradation. Factors such as TGF-β and insulin-like growth factor-1 (IGF-1) secreted by MSCs promote the synthesis of collagen and fibronectin, enhancing structural integrity (Burke et al., 2011; Murata et al., 1997). Simultaneously, MSCs regulate the balance between matrix metalloproteinases (MMPs) and their tissue inhibitors (TIMPs). This ensures controlled ECM degradation necessary for trophoblast invasion while preventing excessive breakdown that would damage endometrial structure, maintaining an optimal state for implantation (Wang and Khalil, 2018; Liu et al., 2000; Baker et al., 2002).

#### 4.4.2 Signaling pathway regulation

MSCs fine-tune endometrial cell function by modulating key signaling pathways via paracrine secretion. For instance, MSC-derived hepatocyte growth factor (HGF) can activate the PI3K-Akt pathway in endometrial cells, promoting cell survival and proliferation (Zhou et al., 2014; Le et al., 2016; Xu et al., 2022). Conversely, molecules like Dickkopf-related protein 1 (DKK1) secreted by MSCs can act as Wnt signaling antagonists, preventing aberrant activation and ensuring precise regulation of the implantation process (Hayashi et al., 2011; Tepekoy et al., 2015; Li et al., 2016). Through such coordinated actions, MSCs ensure the endometrial cellular machinery is primed for receptivity.

## 5 Application of mesenchymal stem cells in the treatment of endometrial tolerance-related diseases

When evaluating the clinical applications below, it is important to recognize that improvements in structural parameters—such as increased endometrial thickness, reduced adhesion area, enhanced glandular density, or increased microvessel density—should be viewed as indicators of successful tissue repair. These are necessary but not sufficient conditions for successful implantation. The ultimate therapeutic goal is the restoration of functional endometrial receptivity: a transient, precisely regulated state that enables blastocyst apposition, adhesion, and invasion, and that can only be confirmed by successful embryo implantation and clinical pregnancy. Achieving this requires the integrated action of all mechanisms described in Section 4—structural regeneration, angiogenesis, immunomodulation, and microenvironmental signaling—working in concert with the patient’s endogenous hormonal milieu. This distinction explains why MSC-based therapy, with its multifaceted approach, holds theoretical advantages over single-target interventions, while also clarifying why structural improvements do not invariably translate to pregnancy success. The therapeutic potential of MSCs has been investigated in several clinical scenarios characterized by impaired endometrial receptivity. While preliminary results are promising, it is crucial to evaluate this evidence within the context of current research phases and acknowledge existing limitations. The following sections, supplemented by the summary in Table 2, detail the application of MSCs in intrauterine adhesions, thin endometrium, and recurrent implantation failure.

**TABLE 2:** Overview of representative clinical studies on MSC therapy for endometrial receptivity-related diseases.

Disease type	Cell source and delivery method	Study type/Phase	Key outcomes and findings	References
Intrauterine Adhesions (IUA)	Umbilical Cord MSCs (UC-MSCs) on collagen scaffold, hysteroscopic transplantation	Phase I Clinical Trial	Efficacy: Significant increase in endometrial thickness and restoration of menstrual flow. Clinical pregnancy achieved in some patients	Cao et al. (2018)
Thin Endometrium	Autologous Bone Marrow MSCs (BMSCs), intrauterine infusion	Case Report	Efficacy: A patient refractory to hormonal treatment achieved improved endometrial receptivity and subsequent successful live birth via donor egg IVF after MSC therapy	Patel et al. (2021)
Intrauterine Adhesions	Menstrual Blood-derived MSCs (MenSCs), intrauterine infusion	Pilot Clinical Study	Efficacy: Confirmed feasibility of the protocol. Patients showed increased endometrial thickness and histological improvement in glandular and vascular structures	Zheng et al. (2018)
Recurrent Implantation Failure	Autologous Peripheral Blood Mononuclear Cells (PBMCs), intrauterine infusion	Randomized Controlled Trial	Efficacy: Preliminary evidence suggests potential improvement in endometrial thickness and vascularity, leading to successful implantation in a subset of patients with previous multiple IVF failures	Sheikhansari et al. (2020)

### 5.1 Uterine cavity adhesion

Intrauterine adhesions (IUA), often resulting from damage to the endometrial basal layer, lead to fibrosis and scarring within the uterine cavity, severely compromising fertility (Dreisler and Kjer, 2019; March, 2011). MSC-based therapy aims to regenerate the functional endometrium in these scarred areas.

The most compelling clinical evidence to date comes from a phase I clinical trial by Cao et al., where patients with severe IUA received intrauterine transplantation of umbilical cord-derived MSCs (UC-MSCs) loaded onto a collagen scaffold via hysteroscopy (Cao et al., 2018). This study primarily established the safety of the procedure and reported promising efficacy: significant increases in endometrial thickness, restoration of menstrual flow, and, crucially, the achievement of clinical pregnancy in several patients who had previously been infertile.

However, it is critical to interpret these promising results with appropriate caution. The Phase I trial by Cao et al. (2018), while groundbreaking as the first formal clinical study in this domain, was an uncontrolled single-arm trial with a small sample size (n = 26) and relatively short follow-up duration, precluding definitive conclusions about efficacy and long-term safety. The observed variability in therapeutic response—some patients achieving pregnancy while others showed only modest structural improvement—underscores our incomplete understanding of factors determining MSC engraftment and function within a densely fibrotic microenvironment. Patient selection criteria likely influence outcomes: the extent and chronicity of adhesions, degree of endometrial fibrosis, and prior surgical history may all modulate therapeutic response but remain poorly characterized as predictive variables. Furthermore, the use of a collagen scaffold in this trial complicates attribution of effect—was efficacy driven by MSCs, the scaffold, or their combination? Well-designed randomized controlled trials with appropriate control arms (e.g., scaffold alone, placebo) and standardized outcome measures are urgently needed to establish true therapeutic value and identify optimal candidates.

### 5.2 Thin endometrium

A persistent thin endometrium, refractory to conventional hormonal treatments, remains a significant cause of implantation failure (Zheng et al., 2022). MSCs offer a regenerative strategy beyond mere hormonal stimulation.

Promising evidence, though often from case reports or small series, indicates the potential of MSCs. Patel et al. reported a successful live birth in a patient with a thin endometrium who had failed multiple cycles of hormone replacement therapy (HRT) and *in vitro* fertilization (IVF) (Patel et al., 2021). Following MSC therapy via uterine artery infusion and subsequent HRT, the patient achieved a viable pregnancy through donor egg IVF. This case highlights MSC therapy as a potential intervention for otherwise intractable cases.

Robust preclinical data support these clinical observations. Transplantation of adipose-derived MSCs (ADMSCs) in animal models of thin endometrium has consistently demonstrated significant increases in endometrial thickness, upregulation of angiogenic factors, and improved perfusion (Hong et al., 2023). Nonetheless, translating these findings into predictable clinical success requires further exploration and standardization of critical parameters, including optimal cell dosage, the most effective delivery route, and the precise timing of transplantation within the treatment cycle.

### 5.3 Recurrent implantation failure

Recurrent implantation failure (RIF), defined by the failure to achieve a clinical pregnancy after multiple transfers of good-quality embryos, is often attributed to functional deficiencies in endometrial receptivity (Coughlan et al., 2014). Given their multifaceted mechanism of action, MSCs are being explored as a comprehensive therapeutic option to address the complex pathophysiology of RIF.

Emerging, albeit preliminary, clinical studies have begun to investigate this application. For instance, small-scale exploratory studies have reported that intrauterine infusion of MSCs (e.g., UC-MSCs) in RIF patients can lead to improvements in endometrial thickness and vascularization, with a subset of patients achieving successful implantation and pregnancy in subsequent IVF cycles (Santamaria et al., 2016). This suggests that MSCs may have the capacity to “rejuvenate” a refractory endometrial environment.

It is essential to emphasize that the evidence base for MSC therapy in RIF remains in its infancy and is considerably less mature than for IUA. The studies available are predominantly small-scale, uncontrolled, and heterogeneous in patient inclusion criteria—reflecting the inherent complexity of defining and managing a condition with multiple potential etiologies. The diversity of underlying mechanisms in RIF—ranging from subtle endometrial molecular defects (e.g., altered HOXA10, integrin expression) to systemic immune dysfunction and occult chronic endometritis—means that a “one-size-fits-all” MSC approach is unlikely to be universally effective. The lack of validated predictive biomarkers further compounds this challenge, leading to empirical treatment application and variable success rates. Moreover, the optimal cell type (autologous vs. allogeneic, bone marrow-derived vs. umbilical cord-derived), dose, and timing relative to the frozen embryo transfer cycle have not been systematically investigated. Future investigations must adopt rigorous study designs with well-characterized patient cohorts (ideally stratified by underlying endotype), incorporate placebo controls where ethically feasible, and prioritize the identification of molecular signatures predictive of MSC responsiveness before this approach can be recommended for routine clinical use in RIF.

### 5.4 Potential extension to other conditions: endometriosis

The therapeutic rationale for MSC-based intervention may extend beyond the conditions discussed above to other complex etiologies of impaired endometrial receptivity characterized by chronic inflammation, fibrosis, and immune dysregulation. Endometriosis, affecting up to 10% of reproductive-age women and 30%–50% of those with infertility, represents a compelling candidate for future investigation (Limam et al., 2025). The eutopic endometrium of women with endometriosis exhibits several pathophysiological features that align with the therapeutic mechanisms of MSCs. First, progesterone resistance—a hallmark of endometriosis-associated infertility—results in inadequate secretory transformation and downregulation of implantation-related genes despite apparently normal hormonal levels (Limam et al., 2025). Second, the endometrial immune microenvironment in endometriosis is characterized by altered uNK cell cytotoxicity, M1/M2 macrophage imbalance, and elevated pro-inflammatory cytokines (TNF-α, IL-6)—all contributing to a “hostile” milieu for embryo implantation. Third, even in the absence of overt adhesions, many endometriosis patients exhibit subtle endometrial fibrosis and microvascular abnormalities that may compromise receptivity. Given their well-documented immunomodulatory, anti-inflammatory, and anti-fibrotic properties, MSCs offer a multifaceted strategy potentially capable of addressing these interconnected pathologies. Preclinically, MSCs have been shown to modulate macrophage polarization toward the anti-inflammatory M2 phenotype, suppress TNF-α and IL-6 production, and reduce endometriotic lesion size in animal models through paracrine mechanisms. Recent studies have also explored genetically modified MSCs overexpressing endostatin to inhibit angiogenesis in endometriosis, demonstrating the feasibility of targeted gene therapy approaches. Whether MSCs can reverse progesterone resistance—a conceptually attractive possibility given their hormone-responsive properties (Section 3.1.2)—remains to be investigated.

While direct clinical evidence for MSC therapy in endometriosis-associated infertility remains nascent, the mechanistic overlap with conditions like RIF and IUA provides a strong rationale for future exploration.

If proven effective, MSC-based approaches could offer a novel, fertility-preserving alternative to current hormonal suppression or surgical interventions, which often delay childbearing or compromise ovarian reserve. However, careful consideration of disease stage, endometriotic phenotype, and potential interactions with existing endometriotic lesions will be essential in designing future clinical trials.

## 6 Challenges and bottlenecks in the clinical translation of MSC-based therapy

Despite the promising therapeutic potential of MSCs in restoring endometrial receptivity, their pathway to becoming a standardized clinical treatment is fraught with significant challenges. Addressing these bottlenecks is paramount for the future of the field. This section delineates the key limitations that currently hinder the widespread clinical application of MSCs in reproductive medicine.

### 6.1 Long-term safety and tumorigenicity concerns

While MSCs are generally considered safe in the short term due to their low immunogenicity and no severe adverse events reported in initial trials, their long-term safety profile remains incompletely defined. The primary concerns stem from their self-renewal and differentiation capacities. Although the risk is considered low, the theoretical possibility of ectopic tissue formation or unintended differentiation exists. More notably, the potential for promoting tumor growth, either through direct interaction with occult malignant cells or by facilitating angiogenesis in a pre-malignant lesion, warrants rigorous, long-term follow-up in clinical participants (Barkholt et al., 2013). Current studies are often limited in duration, underscoring the need for extended surveillance and robust reporting systems.

### 6.2 Suboptimal delivery and low engraftment efficiency

A major translational hurdle is the inefficient delivery and poor retention of MSCs at the target site. Common routes like intrauterine infusion are simple but may result in significant cell loss due to menstrual fluid reflux or rapid clearance. While direct injection under hysteroscopy may improve localization, it is more invasive. Furthermore, a hostile endometrial microenvironment in severe pathology—characterized by inflammation, fibrosis, and hypoxia—can lead to massive cell death post-transplantation, severely limiting the durable engraftment necessary for sustained therapeutic effect (Heissig et al., 2015).

### 6.3 Incomplete understanding of mechanisms and predictive biomarkers

Although MSCs are known to act through multiple mechanisms (paracrine, differentiation, etc.), the relative contribution of each pathway in achieving clinical success in different endometrial pathologies remains unclear. This knowledge gap makes it difficult to rationally optimize therapy (Lee et al., 2011). Consequently, there is a critical absence of validated biomarkers to predict which patients will respond to MSC therapy. Treatment is currently applied empirically, leading to variable success rates and unnecessary costs for non-responders.

### 6.4 High costs and regulatory hurdles

The translation of MSC therapy from the bench to the bedside is an expensive endeavor. Costs associated with Good Manufacturing Practice (GMP)-compliant cell production, quality control, storage, and transportation are substantial. These high costs, combined with the current lack of robust Phase III trial data demonstrating unequivocal efficacy, pose significant challenges for healthcare reimbursement systems. Furthermore, navigating the complex and evolving regulatory landscape for cell-based products requires significant time and resources, slowing down clinical development (Mendicino et al., 2014).

## 7 Conclusion

Mesenchymal stem cells (MSCs) represent a transformative therapeutic strategy for addressing impaired endometrial receptivity (ER), leveraging a synergistic combination of cellular differentiation, paracrine-mediated angiogenesis, immunomodulation, and microenvironmental remodeling. This review has synthesized compelling evidence demonstrating that MSCs function not merely by replacing damaged cells but by orchestrating a holistic restoration of the endometrial niche, making them uniquely positioned to treat conditions where conventional hormonal therapies often fail.

The clinical translation of this approach, while still in its nascent stages, shows considerable promise. Preliminary applications in intrauterine adhesions (IUA) and thin endometrium have yielded encouraging outcomes, including statistically significant increases in endometrial thickness and the restoration of fertility in previously refractory cases. For instance, clinical studies have reported pregnancy successes following MSC administration in patients with mean endometrial thickness increasing from critically low levels to within the receptive range.

However, the path to clinical routine is fraught with challenges that must be rigorously addressed. Key bottlenecks include the long-term safety profile of cell transplants, the critical lack of standardized protocols for cell production and delivery, and the significant variability in individual patient responses. The high cost of therapy further presents a barrier to widespread accessibility.

Future perspectives should strategically focus on several key areas to advance the field. The development of precision medicine approaches is needed to identify optimal candidates and tailor treatments. Concurrently, the exploration of cell-free therapies utilizing MSC-derived exosomes as safer, off-the-shelf alternatives is warranted. Furthermore, the engineering of advanced biomaterial scaffolds will be crucial to enhance cell retention and survival. Finally, the execution of large-scale, randomized controlled trials is essential to conclusively establish efficacy and safety. Through interdisciplinary collaboration and sustained research efforts, MSC-based therapies hold the definitive potential to revolutionize the management of infertility and fundamentally improve outcomes for patients worldwide.
